# Portable Power Generation for Remote Areas Using Hydrogen Generated via Maleic Acid-Promoted Hydrolysis of Ammonia Borane

**DOI:** 10.3390/molecules24224045

**Published:** 2019-11-08

**Authors:** Taylor B. Groom, Jason R. Gabl, Timothée L. Pourpoint

**Affiliations:** 1 School of Mechanical Engineering, Purdue University, West Lafayette, IN 47906, USA; tgroom@purdue.edu; 2 School of Aeronautics and Astronautics, Purdue University, West Lafayette, IN 47906, USA; jgabl@purdue.edu

**Keywords:** hydrogen fuel cells, portable power production, ammonia borane hydrolysis, acid-promoted hydrolysis

## Abstract

A significant drawback to ammonia borane as a hydrogen storage material is the production of ammonia gas during hydrolysis. As a possible solution, maleic acid is shown to be capable of fully promoting hydrolysis of ammonia borane while also preventing ammonia release in excess of single digit parts per million. The reaction is shown to be relatively insensitive towards common water contaminants, with seawater, puddle water, and synthetic urine resulting in hydrogen evolution comparable to that observed when using highly pure deionized water. A common cola beverage was also investigated as a potential water source, with results deviating from those observed when using the other water sources. The ability to use low quality water sources presents the option of acquiring water at the point of use, greatly increasing the energy density of the system during transportation. For each of the water sources being used, concentrations of ammonia in the gas products of maleic acid-promoted hydrolysis were found to be less than the lower detection limits of the employed analysis methods. Based on this reaction, a portable hydrogen reactor is reported and shown to be capable of on-demand hydrogen generation sufficient to power a proton exchange membrane fuel cell at varying loads without significant changes in system pressure. The overall power production system has substantial value in scenarios where electrical power is required but there is no access to an established electrical utility, with prime examples including disaster relief and expeditionary military operations.

## 1. Introduction

Over the course of several decades, ammonia borane has been extensively investigated as a hydrogen storage material for various applications. There are multiple methods for releasing hydrogen from ammonia borane, with hydrolysis being advantageous for portable applications because it can be conducted at room temperature and can be scaled to meet a wide range of hydrogen consumption rates. Because ammonia borane is relatively stable in water, a reaction promoter or catalyst is necessary to facilitate hydrolysis at a useful rate [1,2,3]. Many metal catalysts have been reported as being highly active for ammonia borane hydrolysis, with rapid hydrolysis also being observed in acidic solutions. The investigations of acid promoted hydrolysis are most significant to this present work as they provide the basis for the portable power system being reported.

In 1979, Kelly and Marriot proposed two mechanisms to describe potential routes by which an acidic solution could promote hydrogen release from ammonia borane [4]. In 2007, Stephens et al. used reaction rates and isotope effects to determine that that the most likely of the two mechanisms proposed by Kelly and Marriot is protonation of the nitrogen atom to form ammonium and borane, the latter of which is rapidly hydrolyzed to release hydrogen gas. In aqueous solutions, ammonia and ammonium exist in a pH-dependent equilibrium, with low pH conditions favoring ammonium. When using maleic acid to promote ammonia borane hydrolysis, the reaction environment is an acidic solution with a low pH. This means the ammonium formed when the nitrogen atom in ammonia borane is protonated will stay in solution, rather than form ammonia and escape from the solution with the evolved hydrogen. This is the fundamental benefit of acid promoted hydrolysis compared to metal-catalyzed hydrolysis as it has the potential to form a single step hydrogen generation and purification scheme.

Building on the literature cited above, previous studies by our group have shown organic acid-promoted hydrolysis of ammonia borane to result in the release of highly pure hydrogen gas that can be used to power proton exchange membrane fuel cells (PEMFCs) [5]. In addition to reporting reaction kinetics, hydrogen yield, and hydrogen purity when using highly pure deionized water, our previous paper presented initial results that indicate low-quality water sources can be used to promote hydrogen release with reaction rates and yields similar to those achieved when using high purity water sources. These low-quality water sources include puddle water, seawater, and a synthetic urine surrogate. A thorough hydrogen purity analysis is included in the present paper in order to verify that hydrogen generated when using these water sources is pure enough to fuel a PEMFC without performance degradation.

Based on the work mentioned above, we propose a portable power generation system which uses hydrogen generated via organic acid-promoted hydrolysis of ammonia borane to power a PEMFC. Such a system would be useful in remote locations or in areas where an established electrical utility is not available, with envisioned use cases including disaster relief, first responders, and expeditionary military operations. The utility of such a system is derived from the ability to use low quality water sources to promote hydrogen release, which permits for the water required for the reaction to be collected from in situ resources at the point of use. Because water is procured at the point of use, the system’s energy density during transportation is much higher than when enough water to promote the reaction and fully dissolve all reactants and byproducts must also be stored with the system. However, because the envisioned end-use cases are highly risk-averse, it is imperative that the reaction kinetics and hydrogen yield are relatively indifferent to the water source being used and that the hydrogen evolved when using low-quality water sources is highly pure and free of detrimental levels of contaminants that could damage a PEMFC. An emphasis of this work is to report on the impact that low-quality water sources have on the purity of hydrogen generated via ammonia borane hydrolysis, as well as to demonstrate a system prototype comprised of a custom hydrogen reactor and a commercially available PEMFC. A detailed explanation of all test methodologies, as well as sourcing for all chemicals and equipment used during experimentation, are included in Section 4 of this paper.

## 2. Results

### 2.1. Rate and Yield of Hydrogen Generation

Our previous report on organic acid-promoted hydrolysis of ammonia borane included rates and yields of hydrogen generation when using either tartaric acid or oxalic acid to promote hydrolysis. While both of these acids were capable of promoting hydrolysis with favorable results, for this paper we have chosen to use maleic acid to promote hydrolysis as it is more water-soluble than oxalic acid and its first pKa is lower than that of tartaric acid (1.94 compared to 2.89). Water solubility is an important factor for system design as a more soluble acid will require less water to become fully dissolved which allows for a more compact reaction vessel. Previous authors have shown the kinetics of acid promoted hydrolysis to be first order with pH, meaning for a given molarity of solution, an acid with a lower pKa will lead to more rapid hydrolysis [4]. Additionally, maleic acid is cheaper than either tartaric acid or oxalic acid, making it attractive for commercial applications.

**Figure 1:**
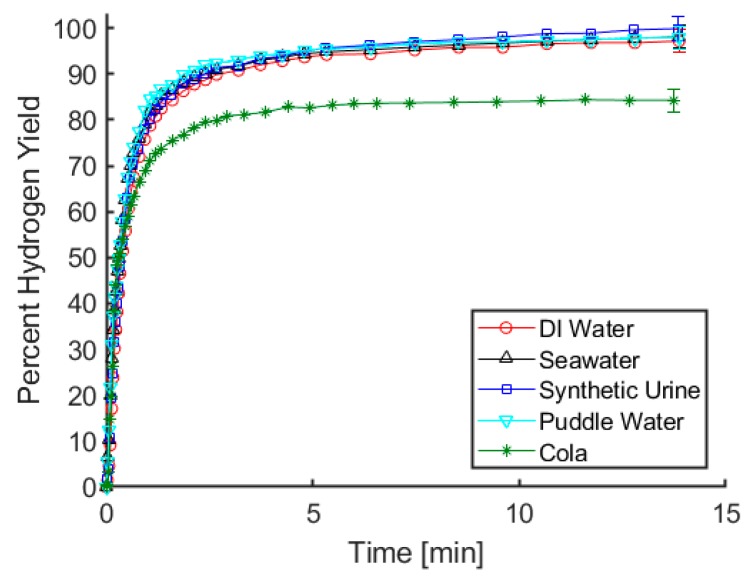
Hydrogen yield using maleic acid and a variety of water sources to promote ammonia borane hydrolysis. Reactions were conducted at room temperature and were initiated by injecting 0.75 mL of 2 M acid solution into 5 mL of approximately 0.3 M ammonia borane solution, with both solutions being made using the indicated water source.

An important trait for our proposed hydrogen reactor is that for any given water source, the rate and yield of hydrogen release remain relatively constant. As can be seen in Figure 1, there is virtually no difference in rate or yield of gas evolution when using maleic acid and deionized water, seawater, synthetic urine, or puddle water. Tests using a carbonated cola beverage resulted in an initial rate of hydrogen release that was comparable to the results using other fluids, but only 84% of the theoretical hydrogen yield was observed. It is notable that gas evolution occurred as soon as ammonia borane and cola were mixed, even prior to acid addition. Considering that the cola has a pH below 3, it is likely that the acidity of the soft drink resulted in the generation of some amount of hydrogen upon initial contact with ammonia borane. Using our test procedures, there is a brief moment between mixing the cola with the ammonia borane and connecting the vessel containing the solution to the gas measurement apparatus, and it is likely that the lower hydrogen yield can be attributed to gas generation during that time. From an application point of view, using the cola resulted in a great deal of foaming and left a sticky residue on the glassware used for these tests. While foaming solutions and sticky glassware are only mild nuisances in a laboratory setting, in a fielded system these factors could potentially lead to clogged tubing or moving parts becoming stuck. For these reasons, carbonated soft drinks such as cola are not an ideal water source for hydrolysis. For the remaining water sources, the results in Figure 1 indicate that at least with respect to rate and yield of gas evolution, acid-promoted hydrolysis is relatively indifferent to the water source being used.

### 2.2. Hydrogen Purity

**Figure 2:**
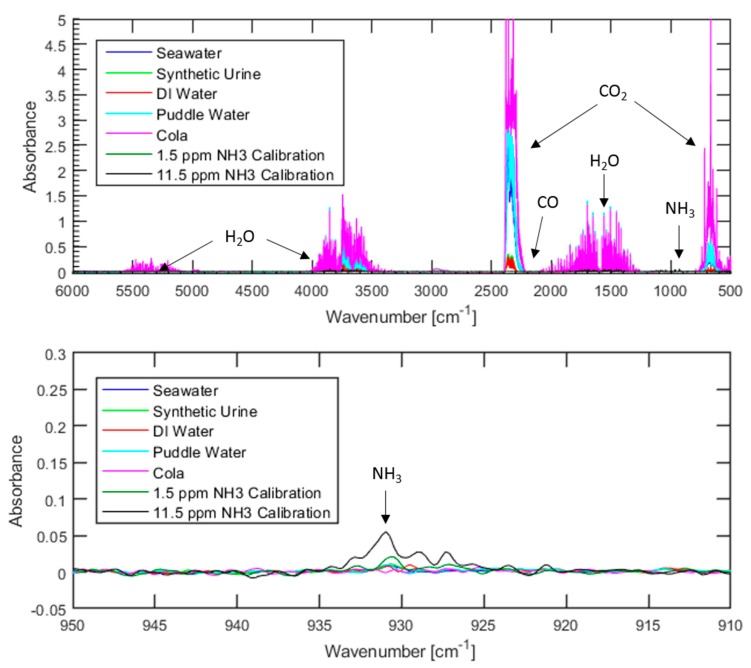
FTIR spectrums of gases evolved from organic acid-promoted ammonia borane hydrolysis using various water sources. The top figure shows the entire spectrum while the bottom figure shows a detailed view of the region where an ammonia response would be found.

To ensure the PEMFC compatibility of hydrogen generated via organic acid-promoted hydrolysis of ammonia borane using low-quality water sources, Fourier transform infrared spectroscopy (FTIR) was used to detect contaminants within the gas products. Three contaminants were of particular interest: ammonia, because it is a byproduct of metal-catalyzed ammonia borane hydrolysis and is highly damaging to PEMFCs; carbon monoxide because it is also highly damaging to PEMFCs, and carbon dioxide because it readily dissolves in water and can be found in many water sources. Both ammonia and carbon monoxide have been found to be damaging to fuel cells at levels as low as single-digit parts per million, with specific examples of the extent of fuel cell degradation varying based on fuel cell design and operational parameters [6,7,8,9,10]. While the influence of carbon dioxide contamination is not as severe at low concentrations, several reports have found gas mixtures that contain between 10–50% (100,000–500,000 ppm) of carbon dioxide to be damaging to fuel cells [6,7,11]. Gas analysis tests were conducted using the same water sources that were used for comparison of the hydrogen generation rate and yield. The resulting spectrums, as well as two spectrums from the ammonia calibration curve that have been added for comparison, can be seen in Figure 2 below

Regardless of the water source used, ammonia levels were below the 7.4 ppm lower detection limit associated with these conditions. Similarly, none of the tests resulted in carbon monoxide levels above the lower detection limit of 19.3 ppm. However, carbon dioxide was detected in each spectrum, with the concentration being highly dependent on the water source that is used. Not surprisingly, the cola resulted in the highest carbon dioxide levels with over 2500 ppm, while deionized water led to levels less than 300 ppm. Considering that previous studies concerning the influence of carbon dioxide on fuel cell performance typically investigate carbon dioxide concentrations of 100,000 ppm or greater, it is highly unlikely that 2500 ppm or less of carbon dioxide would lead to fuel cell degradation. A summary of the results of FTIR testing can be seen in Table 1 below.

Because ammonia can damage fuel cells at concentrations below the 7.4 ppm lower detection limit of the FTIR tests discussed above, further testing was conducted using Dräger ammonia detection tubes capable of measuring ammonia levels as low as 0.25 ppm. Hydrolysis was conducted under similar conditions to those used during FTIR testing, with solutions being made using deionized water. The test was repeated several times, with the Dräger tubes confirming the absence of ammonia in excess of 0.25 ppm.

The presence of carbon dioxide in the tests with puddle water and seawater is expected as both are exposed to open air for long periods of time prior to use, allowing for carbon dioxide from the atmosphere to dissolve into the water. The solubility of carbon dioxide in water decreases as pH decreases, meaning that when acid is injected into the system the equilibrium between aqueous and gaseous carbon dioxide will shift to favor the gas. It is highly likely that carbon dioxide dissolved in the puddle water and seawater prior to use is responsible for the presence of carbon dioxide in the spectrum in Figure 2. This hypothesis is further supported by the very low levels of carbon dioxide detected when using deionized water or synthetic urine, both of which have minimal exposure to open-air prior to use.

**Figure 3:**
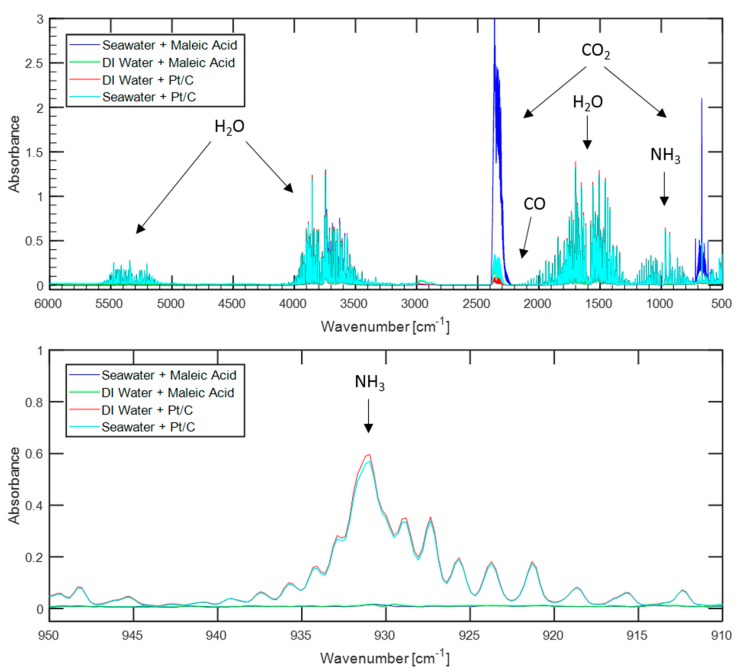
Comparison of FTIR spectrums of gases evolved from either maleic acid-promoted ammonia borane hydrolysis or platinum-catalyzed ammonia borane hydrolysis. The top figure shows the entire spectrum while the bottom figure shows a detailed view of the region where an ammonia response is found.

In order to directly compare the influence of water sources on hydrogen purity when using organic acids versus metal catalysts, similar tests to those discussed above were conducted by replacing the maleic acid with 10 wt.% platinum on a carbon support to catalyze hydrolysis. The platinum to ammonia borane loading was approximately 0.018:1 by mass, which is the same loading ratio as used by previous authors [12]. Tests were conducted using seawater and deionized water, with the gas products being analyzed by FTIR. The resulting spectrums can be seen in Figure 3, along with those from maleic acid promoted hydrolysis using the same water sources for ready comparison.

Using the platinum-catalyst resulted in approximately 1100 ppm of ammonia when using either seawater or deionized water, far more than the non-detectable amount (less than 7.4 ppm) evolved when using maleic acid. Such high levels of ammonia would necessitate a further ammonia sequestration system before hydrogen evolved via platinum-catalyzed hydrolysis could be used to power a PEMFC.

Lower levels of carbon dioxide were detected in the product gas stream when using the platinum catalyst than when using maleic acid. Using deionized water, the gas sample from platinum catalysis contained less than 26 ppm of carbon dioxide, while using maleic acid and the same water source resulted in nearly 300 ppm. Similarly, using platinum and seawater resulted in 350 ppm of carbon dioxide compared to 1700 ppm when using maleic acid. These results strengthen the argument that carbon dioxide enters the system dissolved in the water sources and is primarily released when the pH of the water source is lowered during acid addition.

The water source used for hydrolysis does not seem to have an influence on the levels of carbon monoxide or ammonia that are released during hydrolysis, but does have an influence on the levels of carbon dioxide that are released. The choice of reaction promoter does have a strong influence on the levels of ammonia and carbon dioxide detected in the hydrolysis gas products, with platinum catalysts resulting in high levels of ammonia and low levels of carbon dioxide. Carbon monoxide was never measured in excess of the 19.3 ppm lower detection limit for any of the combinations of water sources and reaction promoter that were investigated.

### 2.3. Hydrogen Reactor Design

**Figure 4:**
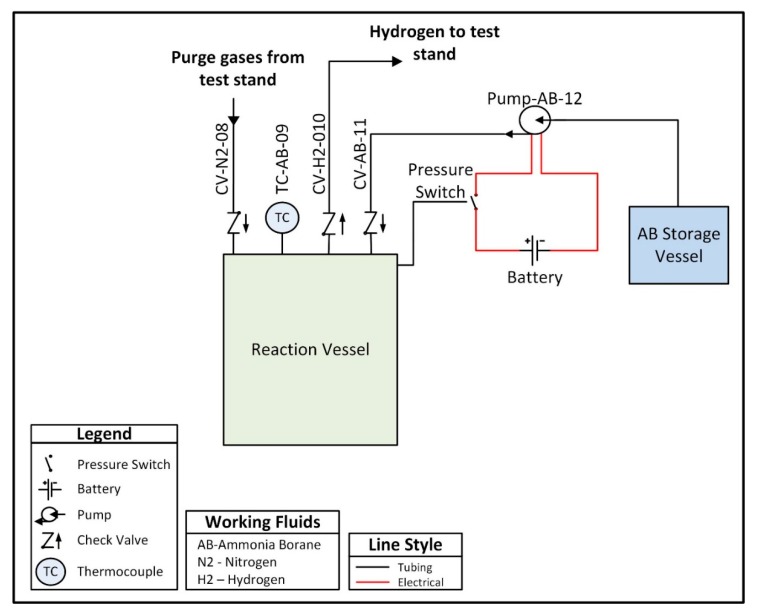
Plumbing and instrumentation diagram (PandID) of the hydrogen reactor used to provide on-demand hydrogen generation.

A custom hydrogen reactor was designed to accommodate acid promoted hydrolysis, and is comprised of two chambers connected by a peristaltic pump. One chamber, referred to as the ammonia borane chamber, is a lightweight vessel that houses an ammonia borane solution and is not subjected to internal pressure. The second vessel, referred to as the reaction chamber, houses an acid solution and is designed to withstand pressures up to 200 kPa. The peristaltic pump is used to move the solution from the ammonia borane chamber into the reaction chamber in order to initiate hydrolysis and generate hydrogen. A check valve between the reaction chamber and the peristaltic pump prevents fluid from the reaction chamber from back flowing into the line between the two chambers. The pump is intermittently powered using a 9 V battery and is controlled using a normally closed pressure switch which opens to break the circuit when the internal pressure exceeds 150 kPa, thus stopping the flow of ammonia borane solution and preventing a buildup of hydrogen pressure. The low-pressure switch is a passive control unit, meaning the only electrical input required is the power supply (in this case the 9 V battery) for the peristaltic pump. In future prototypes, the pump could be powered by the fuel cell once the system has reached steady-state operation, with an external power source only being required during the startup transient. The entirety of the hydrogen reactor and the control system is approximately 500 g. A plumbing and instrumentation diagram (PandID) of the reactor is shown in Figure 4 below.

The design of this system creates a passively controlled, on-demand hydrogen generation scheme that can provide a wide range of gas evolution rates as required by the fuel cell to meet the load demand. This allows the system to operate at varying loads without wide fluctuations in hydrogen pressure. This is an important factor with regards to the safety of the system, especially when considering risk mitigation for the potential use case of military operations where the vessel could be compromised while filled with hydrogen gas. Operating at low pressures also allows for thinner vessel walls and lighter hardware which increases the energy density of the system.

### 2.4. Power Production Testing

Using the hydrogen reactor detailed above and the test stand detailed in Section 4.4, acid-promoted hydrolysis of ammonia borane was used to generate hydrogen and power a PEMFC in order to validate our proposed portable hydrogen generation system. Tests were conducted using 7.5 g of ammonia borane and 28.2 g of maleic acid, providing a 1:1 molar ratio of the two. The ammonia borane was dissolved into 30 g of water to form a 20 wt.% solution, while the maleic acid was dissolved into 145 g of water. The pressure switch was set to 152 kPa and the pressure regulator upstream of the fuel cell was set to 145 kPa, per the fuel cell manufacture’s specifications. The electronic load controller was set to draw constant current loads of 2 A for ten minutes, followed by ten minutes each of 3 A, and 4 A. Prior to the test, the fuel cell was operated using a facility hydrogen supply to allow it to reach steady-state temperature and operation prior to switching over to hydrogen released from ammonia borane.

**Figure 5:**
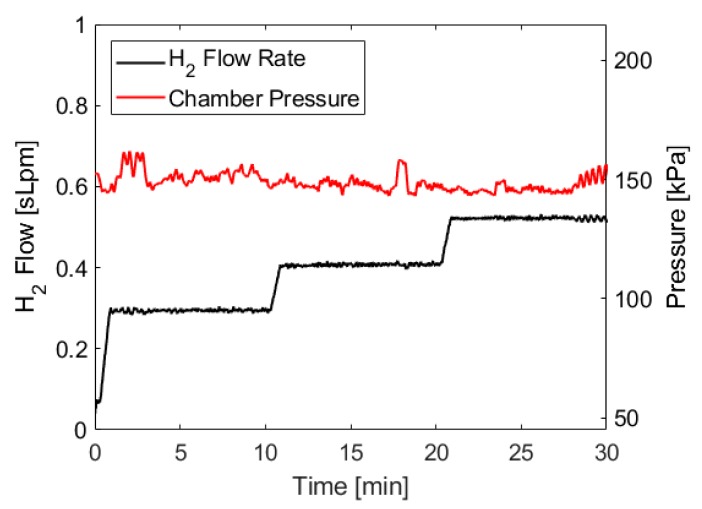
Pressure and flow rate data when using ammonia borane hydrolysis to power the proton exchange membrane fuel cells PEMFC and supply a 2 A, 3 A, and 4 A load, each for 10 min. Hydrogen was generated using approximately 35 mL of 8 M ammonia borane solution and 200 mL of 1.4 M maleic acid solution, both initially at room temperature. The molar ratio of ammonia borane to maleic acid was 1:1. Ammonia borane solution was introduced to the acid solution at a rate dependent on the fuel cell’s hydrogen consumption.

The pressure trace and hydrogen flow rate for the test described above are shown in Figure 5. Because of the water management strategies employed by the fuel cell (described in Section 4.4, the raw pressure trace and flow rate data have brief but significant dips and spikes, respectively, so the data shown below has been smoothed across 30 s moving intervals in order to show the average chamber pressure and flow rate and make it easier to identify pressure trends. The 30 s interval averages three cycles of water management events. These smoothed traces are more representative of what would be observed with a fuel cell stack that uses different water management strategies.

Figure 5 shows a relatively consistent pressure trace throughout the test, with notable deviations occurring at the beginning and at the end of the test. Initially, there is a brief pressure spike as the system ramps up to steady-state conditions. This behavior has been observed in all tests conducted with this apparatus, as there is a slight delay between ammonia borane solution being added to the reaction vessel and enough hydrogen being generated to build up sufficient pressure to engage the pressure switch. The duration of the startup transient is dependent on the volume of the reaction chamber and could be shortened by limiting the headspace in the reactor, which would also result in more frequent cycling of the pressure switch. At the end of the test, the ammonia borane solution pumping cycles become longer as the reaction kinetics slow due to decreasing acid concentration, similar to the behavior observed near the endpoint of acid-base titrations. This causes the pump to operate longer during each cycle in order to add enough ammonia borane to compensate for slower reaction kinetics, which leads to more exaggerated pressure fluctuations. Again, this behavior is consistent across all tests we have conducted. However, the duration of the fluctuating period at the end of the test does not appear to scale with the duration of the test, meaning approximately the same duration of increased fluctuations were observed during a 10 min test as a 30 min test.

**Figure 6:**
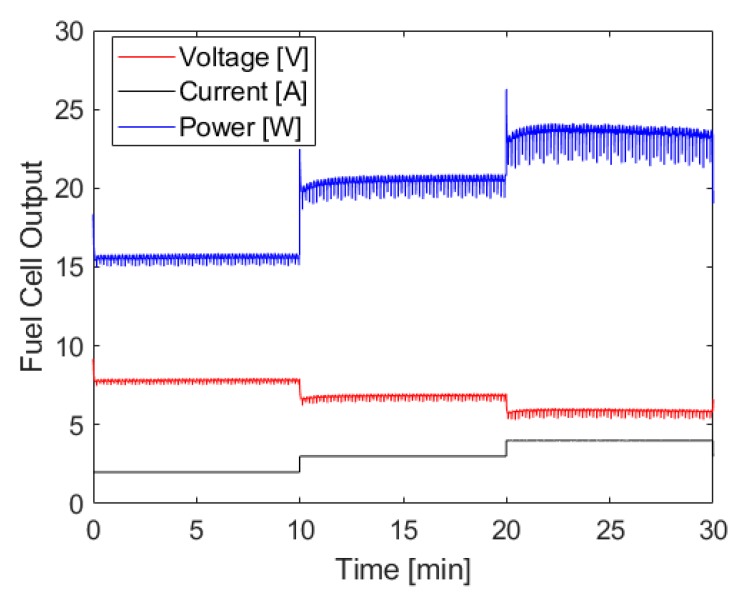
Fuel cell current, power, and voltage output when using ammonia borane hydrolysis to power a PEMFC. Hydrogen was generated using approximately 35 mL of 8 M ammonia borane solution and 200 mL of 1.4 M maleic acid solution, both initially at room temperature. The molar ratio of ammonia borane to maleic acid was 1:1. Ammonia borane solution was introduced to the acid solution at a rate dependent on the fuel cell’s hydrogen consumption.

The fuel cell output generated using the hydrogen generation shown above can be seen in Figure 6, and shows the hydrogen generation system’s ability to power the fuel cell at various loads without an associated increase in chamber pressure. There are brief voltage spikes whenever the load controller shifts from one current setting to another, which is due to the capacitor discharging down to the operating voltage of the fuel cell at the new current level. There are also small fluctuations in voltage and power that are due to the water management strategy of this fuel cell. Outside of these exceptions, the power output at each level is quite constant with only a slight decrease at the tail end of the test which aligns closely with the fluctuations in pressure described above. When averaging the power output across 30 s moving intervals to smooth power drops due to water management events, the standard deviation of power at each step of constant current demand is less than ± 0.1 W.

**Figure 7:**
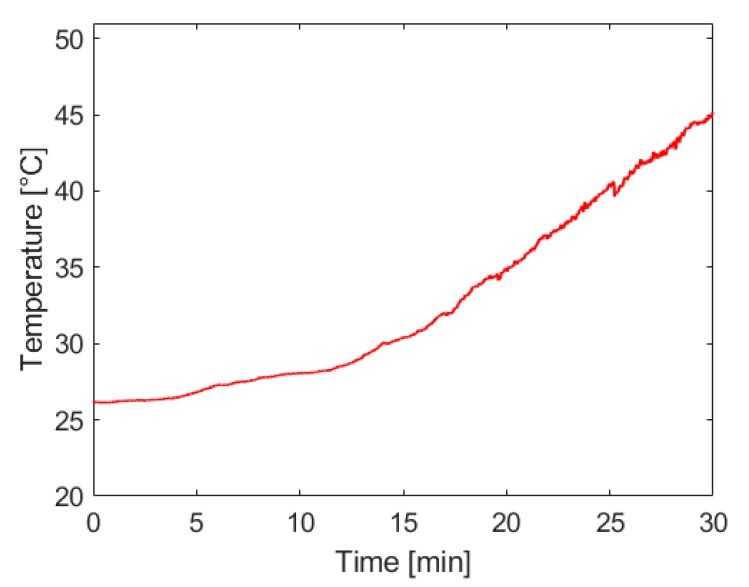
Temperature inside the reaction vessel used to house maleic acid-promoted hydrolysis of ammonia borane. Hydrogen was generated using approximately 35 mL of 8 M ammonia borane solution and 200 mL of 1.4 M maleic acid solution, both initially at room temperature. The molar ratio of ammonia borane to maleic acid was 1:1. Ammonia borane solution was introduced to the acid solution at a rate dependent on the fuel cell’s hydrogen consumption.

Figure 7 below shows that the rate of temperature rise in the reaction vessel increases with the rate of hydrogen generation, as would be expected from this exothermic reaction. The most significant concerns associated with system temperature rise are the potential of injuring the system operator and an increase of water vapor entrained in the hydrogen being supplied to the fuel cell. Future work on this system will include the introduction of passive heat management techniques that can maintain a safe operating temperature and extend the operational capacity of the system.

## 3. Discussion and Conclusions

The results of this study can be categorized into two parts, the first serving to validate that highly pure hydrogen gas can be evolved from organic acid-promoted hydrolysis of ammonia borane when using low-quality water sources, and the second being the demonstration of a system which is built on this concept with many real-world applications in mind. To the first point, it is significant to highlight the difference in hydrogen purity when using maleic acid to promote hydrogen release rather than a platinum-based catalyst. Particularly with respect to ammonia concentration, the hydrogen generated using maleic acid was pure enough to be supplied directly to a PEMFC without further purification, which was not the case when using platinum. The simplicity of a single step hydrogen generation and purification scheme is of great value as it can lower the cost of an end-use system, improve its ease of use, and increase its resiliency towards water contaminants and system malfunctions. However, further investigation of the identification and recyclability of reaction byproducts and environmentally conscious waste disposal will be of utmost importance when considering widespread implementation of such a system.

The second point of this paper is to demonstrate the implementation and real-world value of acid-promoted hydrolysis. The custom hydrogen reactor described in this report is quite simple and could be mass-produced at relatively low cost, and was shown to be capable of supporting on-demand hydrogen generation to fuel a PEMFC at varying load conditions. The energy density of this system compares favorably to current energy storage systems such as lithium-ion batteries. Taking the lower heating value of hydrogen to be 120 MJ/kg, assuming a fuel cell efficiency of 50% and using a 1:1 ratio of maleic acid to ammonia borane, the energy density of the dry reactants is greater than 2400 kJ/kg. The current iteration of the system prototype weighs approximately 1 kg when including the fuel cell, reaction vessel, and all required hardware. If an additional 1 kg of reactants were to be included with the system, the overall energy density would be just over 1200 kJ/kg. For comparison, BB-2590 lithium-ion batteries, widely used by the United States military, have a nominal energy density of 462 kJ/kg or about 2.5 times less than the proposed system. Additional mass savings are possible with optimized manufacturing.

Future work will include longer duration fuel cell testing to ensure prolonged compatibility with PEMFCs. Additionally, we intend to investigate the recyclability of the reaction byproducts.

## 4. Materials and Methods

### 4.1. Materials

Ammonia borane used for measuring reaction yields and kinetics or hydrogen purity was purchased from Sigma-Aldrich (MilliporeSigma, St. Louis, MO, USA, 97% purity, part number 682098) and used as received. Due to the cost of ammonia borane and the relatively large amounts needed for fuel cell testing, ammonia borane used for power production tests was synthesized using the method of Ramachandran and Kulkarni [13]. Maleic acid (>99% purity, part number M0375) and 10 wt.% platinum on an activated carbon support (part number 205958) were purchased from Sigma-Aldrich and used as received. Various water sources were used for this study, including highly pure deionized water, puddle water collected from road water runoff, seawater, Coca-Cola, and a synthetic urine surrogate synthesized in-house and consisting of 94.8 wt.% deionized water, 2.4 wt.% urea (part number U5378), 2.1 wt.% sodium chloride (part number S7653), 0.5wt.% potassium chloride (part number P9333), and 0.2 wt.% creatine monohydrate (part number C3630), all purchased from Sigma-Aldrich.

### 4.2. Burette Tests

Tests measuring reaction yields and kinetics or hydrogen purity were conducted using a 250 mL gas burette setup similar to that described previously by our group [5]. An approximately 2M stock solution of maleic acid was prepared using each of the water sources listed above, and a fresh ammonia borane solution was prepared immediately before each test also using water from one of these sources. For each water source, the initial pH of the acid solutions was between 1.2 and 1.4. Hydrogen generation was initiated by injecting a small amount of the acid solution made with the same water source as the ammonia borane solution, such that only a single water source was used for each test. Unless otherwise noted, all acid-promoted hydrolysis was completed using a 1:1 molar ratio of maleic acid to ammonia borane. The uncertainty in hydrogen yield was approximately 2.5% across all tests, with the uncertainty in reading the gas burette being responsible for the majority of that value. Test conditions were repeated multiple times and found to be highly repeatable within the accuracy of the test apparatus.

### 4.3. Hydrogen Purity Analysis

Fourier transform infrared spectroscopy was conducted using an Agilent Cary 680 FTIR spectrometer (Agilent Technolgoies, Inc., Santa Clara, CA, USA) equipped with a heated gas cell with a 10 m path length (Pike Technologies, Madison, WI, USA). A temperature controller held the gas cell at 150 °C and all measurements were performed with the gas cell at 101 kPa. Samples were loaded into the gas cell by conducting reactions in an adjacent flask and routing the gas products into the gas cell. The line between the reaction flask and the gas cell was placed in an ice bath to condense a fraction of the water vapor entrained in the gas stream such that the concentration of water in the gas cell would not saturate the signal and prevent detection of other contaminants. Prior to initiating reactions, the ammonia borane solution was loaded into the reaction flask and both the flask and the gas cell were purged by repeated evacuation and backfilling using ultra-high purity nitrogen (99.999% pure, part number SG1959112-300) purchased from Indiana Oxygen. With the system partially evacuated to allow for gas generation without over-pressurization, a small amount of acid solution was injected into the reaction flask to initiate hydrogen generation. Following reaction completion, the gas cell was isolated from the reaction flask and brought to atmospheric pressure using ultra-high purity nitrogen. Each sample consisted of approximately 0.016 moles of evolved gases and was scanned 100 times in order to increase the signal-to-noise ratio, which in turn decreased the lower detection limits of the containments. The detected signals were related to concentrations of contaminants using quantitative calibration curves for ammonia (90 ppm, part number 34L-13-90), carbon monoxide (1000 ppm, part number 17L-49-1000), and carbon dioxide (1500 ppm, part number 17L-34-1500) which were created using calibration gases purchased from GASCO (GASCO Affiliates, LLC., Oldsmar, FL, USA)

Using the FTIR configuration described above, the lower detection limit for ammonia is 7.4 ppm. This limit is based on a three to one ratio of signal to noise in the FTIR spectrum. Because ammonia can damage PEMFCs at levels as low as 1 ppm, Dräger ammonia detection tubes (Dräger, Inc., Houston, TX, USA, part number 8101711) were used to measure ammonia concentrations as low as 0.25 ppm in order to ensure PRMFC compatibility. These tests were conducted using the gas burette setup described in Section 4.2, with the Dräger tubes being placed in line between the reaction vessel and the gas burette. For each test, one liter of gas products was passed through the Dräger tubes, per the manufacturer’s specifications. Tests were conducted at room temperature using a 1:1 molar ratio of ammonia borane to maleic acid, with solutions being made using deionized water.

### 4.4. Hydrogen Generation Test Stand

**Figure 8:**
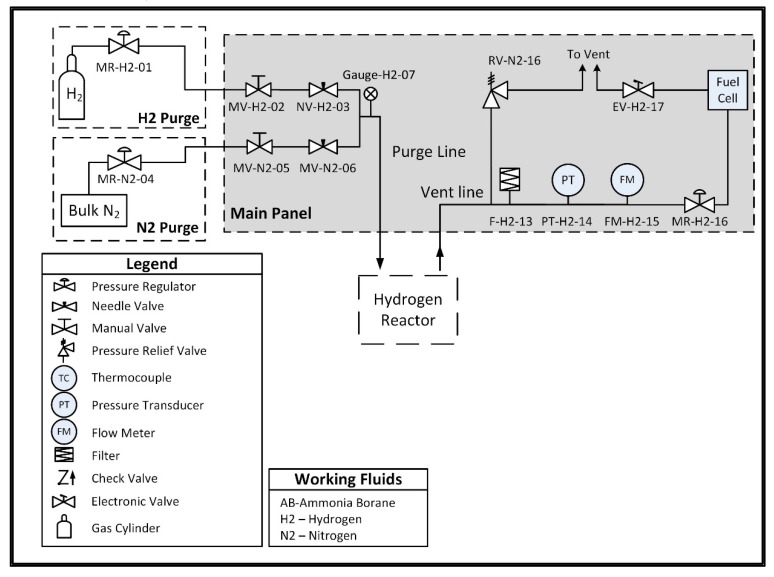
PandID of the hydrogen generation test stand used to conduct hydrolysis and provide hydrogen to the fuel cell.

**Figure 9:**
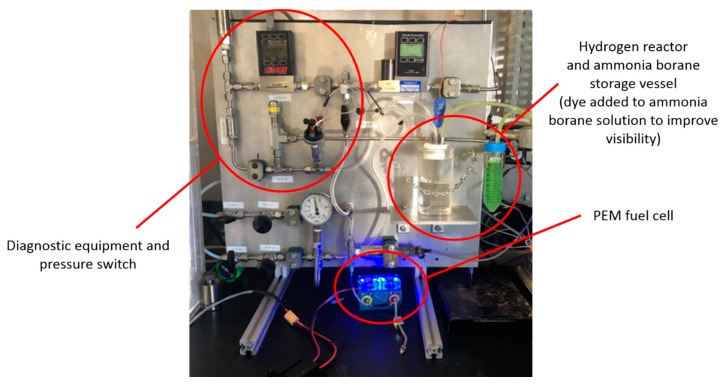
Picture of the hydrogen generation test stand used to conduct hydrolysis and provide hydrogen to the fuel cell.

System-level testing of the hydrogen reactor described in Section 2.3 was conducted using a test stand that allows for monitoring the hydrogen generation reaction and the fuel cell’s power production. The stand is equipped with diagnostic equipment including a Unik 5000 Pressure Transducer (General Electric Company, Boston, MA, USA, 0.2% full-scale accuracy, 0–50 psia range, part number PMP50E6-TB-A1-CA-H0-PE) for measuring hydrogen pressure, a T-type thermocouple, (Omega Engineering, Inc., Norwalk, CT, USA, 1.6 mm × 152.4 mm, ±2 °C accuracy, part number TMQSS-062G-6) for measuring the temperature inside the reaction vessel, and a mass flow meter (Alicat Scientific, Tucson, AZ, USA, accurate to ± 0.01 sLpm ± 0.8% of reading, part number M-5SLPM-D/5M) to measure the hydrogen generation rate. The stand also allows for purging of the reaction vessel with nitrogen and/or hydrogen prior to testing. The reaction vessel is connected to an inline pressure regulator that feeds the fuel cell at a maximum of 145 kPa, per the manufacturer’s specifications. A P&ID of the system is shown in Figure 8 below, and a picture of the stand is shown in Figure 9.

The fuel cell used for this test campaign was a Horizon H-30 Fuel Cell Stack (Horizon Fuel Cell Technologies, Singapore) which is an air-breathing, self-humidified, 30 W PEMFC. This unit operates in a deadheaded configuration using a normally closed electronic valve downstream of the fuel cell. Water management for the H-30 includes a periodic purge event in which the electronic valve is cycled open and closed every ten seconds to allow hydrogen pressure to remove water that is built up inside the stack. Additionally, the unit is self-humidified and generates water vapor using a short circuit event to vaporize water in the stack every ten seconds (out of phase with the purge event) to provide humidification. Both of these events are controlled by a control unit provided by the fuel cell manufacturer.

**Figure 10:**
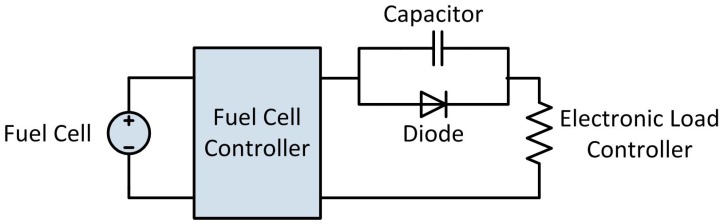
Electrical circuit used for testing the portable power generation system.

Because of the periodic purge and short circuit events, the electrical output of the fuel cell is interrupted every five seconds. In order to smooth these interruptions and provide a more consistent power output, a 2 F, 16 V capacitor was placed in parallel between the fuel cell and the electronic load controller, with a diode preventing back-flow from the capacitor to the fuel cell. There are many literature examples where fuel cell systems which use a short circuit technique to humidify the stack have hybridized the system with a battery in order to provide power to the load during the interruptions, however we chose to use a capacitor to smooth the electrical output while ensuring that all power which is measured by the load controller was produced by the fuel cell and was not supplied by a battery [14]. A diagram of the electrical circuit used for PEMFC testing is shown in Figure 10 below.

An 8600 Series Programmable DC Electronic Load (B&K Precision Corporation, Yorba Linda, CA, USA) was used to provide a constant current load from the fuel cell. Tests were conducted on a series of varying constant current loads to demonstrate the control system’s ability to vary hydrogen generation rates to meet a range of electric power demands without a large change in reactor pressure.

Hydrogen was generated for power production tests by first preparing an ammonia borane solution and a maleic acid solution, with the molar ratio of ammonia borane to maleic acid being 1:1. The solutions were loaded into the hydrogen reactor which was connected to the test stand, and the lines between the ammonia borane chamber and the reaction chamber were primed. The stand and reaction chamber were purged with nitrogen to remove any oxygen in the system, and then subsequently purged with bottled hydrogen. The system was then connected to the fuel cell which was powered for approximately 30 min on bottled hydrogen to allow for the stack to warm up and reach steady-state. With no load applied and the stack operating at open cell voltage, the bottled hydrogen supply was closed and the battery to the peristaltic pump was connected. Once the fuel cell consumed enough hydrogen that the pressure in the reaction chamber dropped below the 145 kPa set point on the low-pressure switch, the pump was powered on and ammonia borane solution was added to the reaction chamber. At this time, the load controller program was initiated such that a load was applied to the fuel cell.

## Figures and Tables

**Table 1:** Hydrogen purity using maleic acid and various water sources to conduct ammonia borane hydrolysis.

Water Source	NH_3_ [ppm]	CO [ppm]	CO_2_ [ppm]
Deionized Water	<7.4	<19.3	291.5
Synthetic Urine	<7.4	<19.3	359.1
Puddle Water	<7.4	<19.3	2065.8
Seawater	<7.4	<19.3	1696.3
Cola	<7.4	<19.3	2502.5
